# Oxidative stress-induced CDO1 glutathionylation regulates cysteine metabolism and sustains redox homeostasis under ionizing radiation

**DOI:** 10.1016/j.redox.2025.103656

**Published:** 2025-04-30

**Authors:** Yumin He, Dan Li, Hongping Ye, Jiang Zhu, Qianming Chen, Rui Liu

**Affiliations:** a State Key Laboratory of Oral Diseases & National Center for Stomatology & National Clinical Research Center for Oral Diseases & Research Unit of Oral Carcinogenesis and Management & Chinese Academy of Medical Sciences, West China Hospital of Stomatology, Sichuan University, Chengdu, 610041, Sichuan, PR China; b Department of Urology, Xindu District People's Hospital of Chengdu, Chengdu, 610500, PR China

**Keywords:** CDO1, Glutathionylation, Oxidative stress, Radiation damage, Cysteine metabolism

## Abstract

Oxidative stress serves as a fundamental mechanism contributing to ionizing radiation-induced damage, which has significant implications for tissue injury. Cysteine dioxygenase type 1 (CDO1) catalyzes the rate-limiting step for cysteine oxidation pathway, thereby playing a crucial role in regulating cellular cysteine availability. However, the regulation of CDO1 activity and cysteine oxidation under ionizing radiation, as well as their subsequent effects on cell viability, remains largely unexplored. In this study, we provide evidence that CDO1 activity and cysteine oxidation are inhibited following radiation exposure. Mechanistically, ionizing radiation-induced oxidative stress triggers glutathionylation of CDO1 at cysteine (C) 164, which impairs CDO1 enzymatic activity by disrupting its interaction with the substrate cysteine. Furthermore, glutathionylation at CDO1 C164 is essential for maintaining cellular redox homeostasis and supports cell viability under ionizing radiation. These findings reveal a novel mechanism through which redox modifications of CDO1 regulate cysteine metabolism and glutathione synthesis under oxidative stress, thereby underscoring its potential as a therapeutic target for addressing radiation-induced injuries.

## 1 Introduction

Oxidative stress represents a fundamental mechanism through which radiation inflicts tissue damage. Ionizing radiation leads to the generation of reactive oxygen species (ROS) through the radiolysis of water and the disruption of the electron transport chain within mitochondria [[1], [2], [3], [4]]. Overproduction of ROS disrupts redox homeostasis, thereby inducing oxidative stress. These highly reactive species engage with critical biomolecules, resulting in DNA damages, lipid peroxidation, and abnormal redox modifications of proteins [3,[5], [6], [7]]. The DNA strand breaks caused by ROS may lead to mutations and genomic instability, which in turn affect gene expression profiles [8]. Lipid peroxidation compromises membrane integrity, thereby hindering vital cellular functions such as signal transduction and organelle dynamics [9]. Oxidative modifications can alter protein conformation, leading to abnormalities in its function, subcellular localization or stability [10,11].

The glutathione (GSH) system plays a pivotal role in cellular redox homeostasis. GSH, synthesized from glutamate, cysteine, and glycine through the concerted actions of glutamate-cysteine ligase (GCLC) and glutathione synthetase (GSS), is renowned as one of the most abundant intracellular antioxidants. Its antioxidant efficacy hinges on the thiol (-SH) group of cysteine, which functions as an electron donor in neutralizing reactive oxygen species (ROS). GSH directly scavenges ROS and also supports GSH-dependent enzymes such as glutathione peroxidases (GPx) and glutathione S-transferases (GSTs) [12]. GPx catalyzes the reduction of hydrogen peroxides and lipid hydroperoxides to their corresponding alcohols, concurrently converting GSH to its oxidized form (GSSG) [13]. GSTs play a vital role in detoxification by conjugating GSH to electrophilic substrates, facilitating their excretion from cells [14,15]. Beyond its antioxidant capacity, GSH regulates protein function through S-glutathionylation, a reversible modification in which GSH covalently binds to cysteine residues on proteins [16]. This process protects proteins from irreversible oxidative damage and serves as a redox-sensitive switch that influences protein activity, structure, and signaling [17]. Additionally, GSH contributes to mitochondrial integrity, immune cell function, and the regulation of cell proliferation and differentiation.

Cysteine, a sulfur-containing amino acid, is implicated in diverse biological processes, including its antioxidant properties, detoxification capabilities, and involvement in protein biosynthesis. The availability of cysteine is widely considered as the rate-limiting factor for GSH synthesis [18]. In addition, the oxidation of cysteine represents another significant metabolic pathway for the utilization of cellular cysteine. Initially, cysteine is converted to cysteine sulfinic acid through the addition of molecular dioxygen to its thiol group, which is catalyzed by cysteine dioxygenase type 1 (CDO1), an iron (Fe^2+^)-dependent thiol dioxygenase, functioning as the rate-limiting step for cysteine oxidation [19]. Subsequently, cysteine sulfinic acid is metabolized by cytosolic aspartate aminotransferase to yield sulfinyl pyruvate, which spontaneously decomposes into pyruvate and sulfite; the latter is further oxidized to sulfate by sulfite oxidase. Alternatively, cysteine sulfinate decarboxylase can transform cysteine sulfinic acid into hypotaurine, which is then oxidized to taurine by hypotaurine dehydrogenase [20]. However, whether cysteine oxidation was regulated in response to ionizing radiation, particularly in the context of oxidative stress, remain largely unexplored. In this study, we demonstrate that CDO1 undergoes glutathionylation at cysteine (C)164 in oral and lung epithelial cells when exposed to radiation, which inhibits CDO1 activity and the cysteine oxidation pathway, ultimately promoting GSH synthesis. Furthermore, the disruption of CDO1 C164 glutathionylation exacerbates radiation-induced oxidative stress and negatively impacts cell viability.

## 2 Materials and methods

### 2.1 Materials

The antibody specific to GST (#2622) and GCL (#48005) was acquired from Cell Signaling Technology. The antibody that recognizes Glutathione (MAB5310) was obtained from Merck. Antibodies targeting Tubulin (ab7291), CDO1 (ab232699), Flag (ab205606), and His (ab18184) were sourced from Abcam. The antibody for S-Nitroso-Cysteine (NBP2-42698) was procured from Novus Biologicals. Additionally, NAC, biotin-maleimide, diamide, and maleimide were purchased from Sigma-Aldrich, and DYn-2 and biotin-azide were obtained from MedChemExpress. [1, 2, 1′, 2′-^14^C] l-Cystine (NEC854050UC) was purchased from Revvity. [1–^14^C] l-Cysteine (MC2362) was purchased from Moravek.

### 2.2 Cell culture and ionizing radiation

BEAS-2B cells were acquired from BIO Biotechnology located in Chengdu, China. These cells were maintained in Dulbecco's Modified Eagle's Medium supplemented with 10 % fetal bovine serum. The HOK cell line was generously provided by Dr. JS Gutkind from the National Institute of Dental and Craniofacial Research in Maryland, USA. HOK cells were cultured in Keratinocyte Serum-Free Medium obtained from Thermo Fisher Scientific, Inc. Cell transfection was performed following previous studies [21,22]. Ionizing radiation was performed using an X-RAD 225 irradiator (Precision X-ray Inc., North Branford, CT) at specified dosage levels.

### 2.3 Immunoprecipitation and immunoblot

Immunoprecipitation and immunoblot was performed according to the previously reported protocol [[23], [24], [25]]. For sodium dodecyl sulfate-polyacrylamide gel electrophoresis (SDS-PAGE), cellular lysis was conducted using a lysis buffer composed of 50 mM Tris-HCl (pH 7.5), 0.1 % SDS, 1 % Triton X-100, 150 mM NaCl, 0.5 mM EDTA, 100 mM phenylmethylsulfonyl fluoride (PMSF), 100 mM leupeptin, 1 mM aprotinin, 100 mM sodium orthovanadate, 100 mM sodium pyrophosphate, and 1 mM sodium fluoride, without the inclusion of reducing agents. Electrophoresis was conducted within 30 min post-lysis. In the case of reducing SDS-PAGE, the lysates were combined with SDS-PAGE loading buffer containing 50 mM Tris-HCl (pH 8.8), 1 % w/v SDS, 2.5 % glycerol, 0.001 % w/v bromophenol blue, and 143 mM β-mercaptoethanol, followed by a 10-min boiling step. In contrast, for non-reducing SDS-PAGE, β-mercaptoethanol was excluded from the gel loading buffer. Immunoprecipitation and immunoblot for the detection of CDO1 glutathionylation was performed following previous studies [26,27]. Briefly, we performed immunoprecipitation using cell lysates with an anti-CDO1 or anti-Tag antibody, and the immunoprecipitation products were then separated by SDS-PAGE followed by immunoblotting against an anti-GSH antibody.

### 2.4 Biotin-labeling of oxidized cysteine

Cells were lysed on ice for 15 min using a lysis buffer containing 50 mM Tris-HCl (pH 7.0), 5 mM ethylenediaminetetraacetic acid, 120 mM NaCl, and 0.5 % Igepal-630, supplemented with protease inhibitors and 100 mM maleimide (Sigma, St. Louis, MO). Following lysis, insoluble materials were eliminated through centrifugation at 20,000×*g* for 10 min at 4 °C, and the resulting supernatant was transferred to a new Eppendorf tube. The protein concentration was quantified using a Bradford assay. SDS was added from a 10 % stock solution to achieve a final concentration of 1 %, and the cell lysates were incubated at room temperature for 2 h with continuous rotation. To eliminate unreacted maleimide, proteins were precipitated by the addition of five volumes of acetone, pre-cooled to −20 °C, and incubated for 20 min at −20 °C. The samples were then centrifuged at 20,000×*g* for 10 min at 4 °C; the supernatants were discarded, and the protein pellet was allowed to air dry completely before proceeding to the next step. The pellet was subsequently resuspended in 200 μl of lysis buffer containing 1 % SDS, 10 mM dithiothreitol (DTT), and 0.1 mM biotin-maleimide (Sigma, St. Louis, MO) to reduce any remaining oxidized sulfhydryl groups and facilitate their reaction with biotin-maleimide. Proteins were again precipitated using five volumes of methanol at −20 °C, as previously described. The dried pellet was then resuspended in 500 μl of lysis buffer and incubated with 10 μl of a 50 % slurry of streptavidin-sepharose beads (GE Healthcare, Piscataway, NJ) followed by rotating at 4 °C for 2 h. The beads were subsequently washed four times with lysis buffer and resuspended in loading buffer for subsequent SDS-PAGE analysis and immunoblotting.

### 2.5 shRNAs and vectors

The short hairpin RNAs (shRNAs) utilized in this investigation were synthesized based on the specified sequences: CDO1 shRNA, TTC TTC TGC AGT AGC TGA G (which targets a non-coding region), and scramble shRNA, GCT TCT AAC ACC GGA GGT CTT. The cDNAs were subsequently cloned into the mock Flag-pcDNA3.1 vector. Mutagenesis was performed using the QuikChange site-directed mutagenesis kit (Agilent).

### 2.6 Protein expression and purification

The expression of wild-type (WT) or mutant CDO1 and Grx1 proteins was conducted utilizing the pCold I or pGEX4T-1 vectors in BL21 (DE3) bacterial strains [28]. Following IPTG induction, the BL21 bacterial cells were incubated for 16 h at either 16 °C or 37 °C, followed by cell lysis.

To purify His-CDO1 proteins, the lysates were subjected to centrifugation, after which the supernatants were applied to a Ni-NTA column (GE Healthcare Life Sciences). The column was subsequently washed with a 20 mM imidazole solution, and the target protein was eluted using a 250 mM imidazole solution. In the case of GST-Grx1 proteins, the lysates were introduced to a GSTrap HP column (GE Healthcare Life Sciences), followed by washing with phosphate-buffered saline (PBS) and elution with a 10 mM reduced glutathione solution. The eluted protein solution was then processed through a HiPrep 16/60 Sephacryl S-200 HR gel filtration column (GE Healthcare Life Sciences) to eliminate contaminants, and the fractions containing the desired protein were collected [29].

### 2.7 CDO1 enzymatic activity assay

The enzymatic activity of CDO1 was assessed using a previously established methodology with slight modifications [30,31]. Briefly, a fresh assay buffer was prepared, consisting of 50 mM MES buffer at pH 6.1, 0.3 mM ammonium iron sulfate, 2.5 mM cysteine and 62.5 uM bathocuproine disulfonate. Equal volumes of the assay buffer and either cell lysate or immunoprecipitation samples were combined and incubated at 37 °C with shaking for 30 min. The reaction was subsequently halted by the addition of 10 % (w/v) trichloroacetic acid. Proteins were precipitated by centrifugation at 10,000 g for 20 min, and 200 μL of the supernatant was transferred to a Dowex minicolumn for elution with water. A volume of 100 μL of the eluate was then mixed with 50 μL of ninhydrin reagent (Sigma Aldrich) and incubated at 90 °C for 10 min, followed by the addition of 150 μL of 95 % ethanol. The absorbance of the resulting mixture was measured at 550 nm. To evaluate the interaction between CDO1 protein and iron, the activity of CDO1 proteins obtained from an *in vitro* kinase assay was analyzed at varying concentrations of EDTA, in accordance with prior reports [32].

To determine the Km value, 0.05 mg/ml purified CDO1 protein was mixed with assay buffer containing 0.1 mM, 0.2 mM, 1 mM, 2.5 mM, 5 mM, 10 mM, 20 mM, 30 mM, 50 mM, or 80 mM cysteine, respectively. Values of Vmax and Km for cysteine were subsequently estimated from the curves of reaction velocity.

### 2.8 DYn-2 probe-based detection of CDO1 sulfenylation

The detection of protein sulfenylation utilizing the DYn-2 probe was conducted in accordance with previously established methodologies [33]. Cells were incubated with 5 mM DYn-2 for 30 min before IR, and then cell were harvested and subjected to three washes with culture medium. The collected cells were subsequently homogenized on ice using sand in conjunction with an extraction buffer composed of 25 mM Tris-HCl (pH 7.6), 15 mM MgCl_2_, 150 mM NaCl, 15 mM pNO_2_PhenylPO_4_, 60 mM β-glycerophosphate, 0.1 % Nonidet P-40, 0.1 mM Na_3_VO_4_, 1 mM NaF, 1 mM phenylmethanesulfonyl fluoride, 1 mM E64, a mixture of 13 Roche protease inhibitors, and 5 % ethylene glycol. The resulting lysates were centrifuged at 16,000 g for 30 min at 4 °C to eliminate cellular debris. The protein concentration in the soluble fractions was quantified using a standard DC Protein Assay (Bio-Rad Laboratories Inc., Hercules, CA). Following the removal of endogenous biotinylated proteins, a click chemistry reaction was conducted with 100 mg of protein for 1 h under rocking incubation at room temperature. The click reaction was subsequently terminated by a 5-min incubation with 1 mM EDTA. Biotin-labeled proteins were then isolated using streptavidin beads and quantified through immunoblot analysis.

### 2.9 *In vitro* protein glutathionylation and deglutathionylation

*In vitro* protein glutathionylation was performed following previous report [34,35]. The WT or mutant CDO1 protein (25 μg/mL) was incubated in a 20 mM HEPES buffer at pH 7.5, supplemented with 1 mM GSH and 0.5 mM diamide, at 20 °C for 30 min.

*In vitro* protein deglutathionylation was conducted in accordance with previously established protocols [36]. Briefly, glutathionylated CDO1 protein was diluted to a concentration of 3 μM within a 300-μl reaction mixture composed of 100 mM Tris-HCl pH 8.0, 2.0 mM EDTA, 1 mM GSH, 0.2 mM NADPH, and 6 μg/mL glutathione reductase. Subsequently, purified Grx1 protein was introduced into the reaction mixture at a concentration of 50 nM and incubated at 30 °C for 2 h.

### 2.10 Measurement for ^14^C-labeled metabolites

To measure the level of cellular ^14^C-pyruvate, pyruvate was firstly converted to acetyl-phosphate by pyruvate oxidase, following previous reports [37,38]. Briefly, after indicated treatment, cells were harvested, homogenized on ice, and centrifuged to remove cell debris. After deproteinization, a total of 20 μL of cell lysate was combined with 500 μL of a reaction buffer composed of 500 μM EDTA, 100 mM potassium phosphate (pH 6.8), 1.0 mM MgSO_4_, 10 μM FAD, 0.2 mM thiamine pyrophosphate, and 0.2 U/ml pyruvate oxidase. This mixture was incubated at 30 °C for 30 min. Subsequently, acetyl-phosphate was isolated utilizing a two-dimensional thin-layer chromatography on 10 cm × 10 cm EMD PEI cellulose-F plates (EMD Chemicals, La Jolla, CA). The first dimension of chromatography was conducted for 45 min using a solvent system consisting of 0.52 M LiCl and 1 % (v/v) glacial acetic acid. After this, the plates were air-dried, incubated in methanol for 15 min, and then air-dried before undergoing a second dimension of development for an additional 90 min. The acetyl-phosphate spot was quantified using a liquid scintillation counter, and the relative level of cellular ^14^C-pyruvate was determined based on the radioactive signal from the acetyl-phosphate. The results were subsequently normalized to the number of cells.

The measurement of ^14^C-labeled total GSH (GSH and GSSG) was performed following previous reports with minor modifications [[39], [40], [41]]. Briefly, following the specified treatment, cells were collected, homogenized on ice, and subjected to centrifugation to eliminate cellular debris. Subsequently, the supernatants underwent deproteinization and were combined with an equilibrium buffer (0.2 M sodium tetraborate-boric acid, pH 9.5) before being heated at 90 °C for 15 min to ensure the complete oxidation of GSH to glutathione disulfide (GSSG) in an alkaline environment. The resulting supernatants were then analyzed using thin layer chromatography, employing an eluent composed of butanol, glacial acetic acid, and water in a ratio of 3:2:1. The GSSG spot was identified with the reference of a standard GSSG visualized through ninhydrin staining. The radioactive signal was subsequently quantified using a liquid scintillation counter and normalized to the cell counts.

#### 2.10.1 Molecular dynamics (MD) simulation

Molecular dynamics (MD) simulation was performed using GROMACS (version 2018.5) to investigate the impact of C164 glutathionylation [[42], [43], [44]]. The topology of the protein was generated using the GROMOS96 54a7 force field, with the SPC water model employed [45]. The CDO1 with C164 glutathionylation was embedded in a cubic periodic boundary box and solvated with explicit water molecules. To mimic physiological conditions, sodium and chloride ions were added to achieve a neutral charge state and a final ionic concentration of 0.15 M. Prior to production simulations, the system underwent energy minimization using the steepest descent algorithm to eliminate unfavorable atomic contacts. The production phase consisted of a 50-ns MD simulation with a 2-fs time step using the leapfrog integrator. Temperature coupling was maintained at 300 K using the Berendsen thermostat. To assess structural stability, the root mean square deviation (RMSD) of backbone atoms was calculated relative to the energy-minimized initial structure.

### 2.11 Colony formation assay

Colony formation assay was performed following previous report [46]. 100 cells were seeded in one well of a 6-well plate. On the second day, the cells were treated with ionizing radiation and then maintained in culture for a duration of 14 days, with the medium being replenished every three days. Subsequently, the colonies were fixed using methanol for 10 min and stained with a 0.05 % (w/v) solution of crystal violet for an additional 10 min. The colonies were then washed five times with phosphate-buffered saline (PBS), and only those colonies containing more than 50 cells were selected for quantification and analysis under a microscope.

### 2.12 Measurement of BrdU incorporation

The assessment of BrdU incorporation was performed utilizing the BrdU Cell Proliferation ELISA Kit (colorimetric) provided by Abcam (catalog number ab126556). Briefly, 3 × 10^4^ cells were seeded into each well of a 96-well plate. On the second day, the cells were treated with ionizing radiation. 12 h later, 20 μl of a diluted 1 × BrdU solution was added to each well, and the mixture was incubated for a duration of 6 h. Upon completion of the BrdU incorporation phase, the cells were fixed, treated with detection antibodies, and the resultant signal was quantified at a wavelength of 450 nm using a microplate reader.

### 2.13 Quantification and statistical analysis

All experiments were conducted a minimum of three times. The data are presented as mean ± standard deviation (SD) and were analyzed using either the two-sided Student's t-test or ANOVA, unless stated otherwise. A p-value of less than 0.05 was deemed statistically significant.

## 3 Results

### 3.1 Ionizing radiation-induced oxidative stress inhibits CDO1 activity

In order to investigate the cysteine oxidation pathway, the oral keratinocyte HOK cell line and the lung epithelial BEAS-2B cell line, both of which were found as sensitive to ionizing radiation [23], were employed as experimental cell models. These cells were subjected to treatment with ^14^C-labeled cystine, a commonly utilized source of cysteine in cell culture medium, and the metabolic flux was assessed by quantifying the production of ^14^C-labeled pyruvate, a byproduct of cysteine oxidation (Fig. 1A). Notably, a significant reduction in the levels of ^14^C-labeled pyruvate was observed following radiation exposure, indicating a repression of cysteine consumption via the cysteine oxidation pathway (Fig. 1B). Consistently, an elevation in cellular cysteine levels was detected in both the irradiated HOK and BEAS-2B cells (Fig. 1C). The radiation-induced accumulation of cellular cysteine does not likely attributable to enhanced uptake of environmental cystine, as similar radioactive signals were observed in the cell lysates of both irradiated and non-irradiated cells following a short-term incubation with ^14^C-labeled cystine (Supplementary Fig. 1). Cellular cysteine availability is the major determinant for the rate of GSH synthesis [47,48]. Indeed, ionizing radiation induced a noticeable increase in the level of ^14^C-labeled total GSH (GSH and GSSG) after HOK and BEAS-2B cells were incubated with ^14^C-cystine (Fig. 1D). These findings suggest that exposure to ionizing radiation inhibited cysteine oxidation and promotes GSH synthesis in oral and lung epithelial cells.

**Fig. 1:**
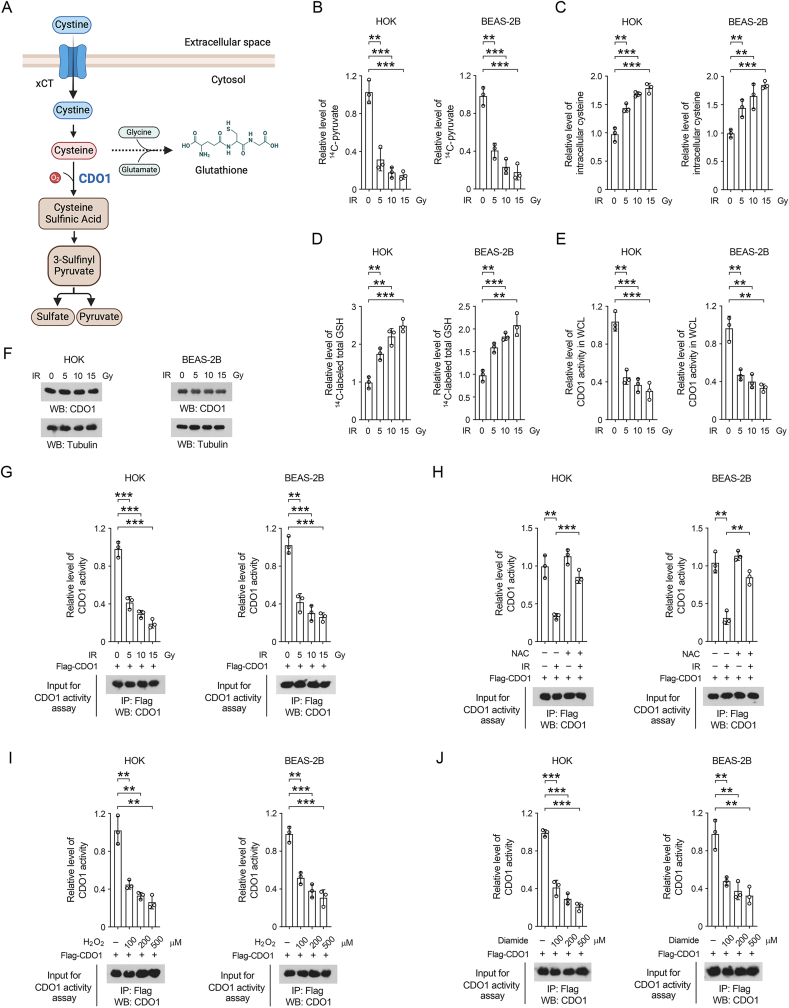
Ionizing radiation-induced oxidative stress inhibits CDO1 activity (B–G) HOK and BEAS-2B cell lines, either with or without Flag-CDO1 expression, were subjected to radiation at indicated doses. (A) A schematic representation of the cysteine oxidation metabolic pathway. (B, D) 1 h after irradiation, cells were incubated with [1, 2, 1′, 2′-^14^C]-cystine (0.1 μCi/ml) for 30 min, and the level of ^14^C-pyruvate and total ^14^C-GSH (GSH and GSSG) were measured. The data are expressed as mean ± standard deviation from three independent experiments. ∗∗*P* < 0.01, ∗∗∗*P* < 0.001. (C) 4 h after irradiation, the intracellular levels of cysteine were evaluated. ∗P < 0.05 and ∗∗P < 0.01. (E) 1 h after irradiation, the enzymatic activity of the CDO1 protein in cell lysates was quantified, with results presented as mean ± SD from three experiments. ∗*P* < 0.05, ∗∗*P* < 0.01, ∗∗∗*P* < 0.001. (F) 1 h after irradiation, immunoblot analysis was performed to assess CDO1 expression, with data derived from three separate experiments. (G) 1 h after irradiation, immunoprecipitation was conducted utilizing the anti-Flag M2 antibody, and the CDO1 activity within the precipitates was analyzed. Results are presented as mean ± SD from three experiments. ∗*P* < 0.05, ∗∗*P* < 0.01, ∗∗∗*P* < 0.001. (H) HOK and BEAS-2B cells expressing Flag-CDO1 were pre-treated with or without 10 mM NAC for 2 h prior to exposure to 10 Gy radiation. 1h after irradiation, immunoprecipitation was performed using the anti-Flag M2 antibody, and the CDO1 activity in the precipitates was evaluated, with results shown as mean ± SD from three experiments. ∗*P* < 0.05, ∗∗*P* < 0.01, ∗∗∗*P* < 0.001. (I–J) HOK and BEAS-2B cells expressing Flag-CDO1 were treated with H_2_O_2_ (I) or diamide (J) at specified concentrations for 30 min. Immunoprecipitation was performed using the anti-Flag M2 antibody, and the CDO1 activity in the precipitates was evaluated, with results presented as mean ± SD from three experiments. ∗*P* < 0.05, ∗∗*P* < 0.01, ∗∗∗*P* < 0.001.

CDO1-mediated cysteine dioxygenation, which utilizes oxygen to produce cysteine sulfinic acid, represents the rate-limiting step in the cysteine oxidation pathway [20]. Exposure to radiation resulted in a 50 % reduction in cysteine dioxygenation activity in cell lysates, which could be observed when cells were irradiated at as low as 5 Gy (Fig. 1E). In contrast, the expression levels of the CDO1 protein exhibited no significant changes (Fig. 1F), suggesting that the observed decrease in cysteine dioxygenation activity was not merely a consequence for alterations in CDO1 protein expression. To investigate the impact of radiation on CDO1 enzymatic activity, equal amounts of Flag-tagged CDO1 protein were precipitated, revealing a significantly reduced activity in CDO1 protein isolated from irradiated cells (Fig. 1G). Notably, pretreatment with N-acetylcysteine (NAC), an antioxidant agent [49,50], substantially prevented the decrease in CDO1 activity in irradiated cells (Fig. 1H). Furthermore, treatment with H_2_O_2_ or diamide was found to inhibit CDO1 activity in non-irradiated cells (Fig. 1I-J). These findings suggest that radiation impairs CDO1 activity and cysteine oxidation pathway in oral and lung epithelial cells, probably through a mechanism involving redox regulation of CDO1.

### 3.2 Ionizing radiation-induced oxidative stress promotes CDO1 glutathionylation at C164

To investigate the regulation of CDO1 in the context of radiation-induced oxidative stress, we assessed the redox status of CDO1 protein. This was accomplished by lysing cells under in the presence of maleimide, which binds and blocks reduced cysteines. Subsequently, proteins were precipitated with acetone to eliminate maleimide. The resuspended proteins underwent treatment with DTT to reduce any remaining oxidized cysteines, which were then labeled with biotin-maleimide for streptavidin precipitation, followed by immunoblotting analyses to quantify the oxidation of cysteine residues. Our findings revealed a significant accumulation of biotin-labeled CDO1 in both HOK and BEAS-2B cells subjected to ionizing radiation (Fig. 2A). These effects were also observed in cells treated with H_2_O_2_ or diamide (Fig. 2B), and could be negated when cells were pretreated with NAC (Fig. 2C), indicating that the cysteine residues of CDO1 were oxidized in response to radiation. Such oxidation of CDO1 cysteine residues was likely reversible, as the addition of DTT after radiation significantly diminished the level of biotin-labeled CDO1 (Fig. 2C). The side chain of cysteine residue in a protein can be oxidized to form various chemical species [51]. Notably, immunoblot analyses utilizing an anti-glutathione antibody revealed a palpably increased signal in Flag-CDO1 precipitates derived from the radiation or diamide-treated cells under non-reducing conditions (Fig. 2D-E), which was mitigated by NAC pretreatment (Fig. 2F), suggesting that CDO1 was glutathionylated. Consistently, by using DYn-2, a probe specific for protein sulfenylation, we pulled down more CDO1 protein from the lysates of irradiated cells, suggesting that ionizing radiation did induce CDO1 sulfenylation, which was considered as an intermediate to form protein glutathionylation (Supplementary Fig. S2A). In contrast, no significant signal was observed with an anti-nitrosylation antibody (Supplementary Fig. S2B). In addition, treatment with diamide did not significantly affect the enzymatic activity of purified CDO1 protein in an *in vitro* system that did not contain GSH or GSSG, implying that CDO1 was unlikely to be oxidized through the formation of disulfide bonds either within a single CDO1 molecule or between multiple CDO1 molecules (Supplementary Fig. S2C). To further support these findings, we established an *in vitro* protein glutathionylation assay by incubating purified CDO1 with GSH and diamide, and detected CDO1 glutathionylation via immunoprecipitation followed by immunoblot analysis with an anti-GSH antibody. This glutathionylation was reversed by treatment with DTT or 2-Mercaptoethanol (2-ME) (Fig. 2G). Protein deglutathionylation was primarily catalyzed by Grx1, utilizing glutathione as a cofactor [52]. As expected, glutathionylation of purified CDO1 proteins, introduced by mixture with GSH and diamide, was abolished upon incubation with purified WT Grx1 protein, but not with the loss-of-function Grx1 C23S mutant (Fig. 2H) [36]. In addition, either ionizing radiation or diamide induced similar level of glutathionylation between WT Flag-CDO1 and Flag-CDO1 R60A (Supplementary Fig. S2D), a mutant with reduced substrate cysteine-binding affinity [53], suggesting that association with the substrate cysteine was not likely to affect CDO1 glutathionylation. These results indicated that radiation-induced oxidative stress promotes CDO1 glutathionylation.

**Fig. 2:**
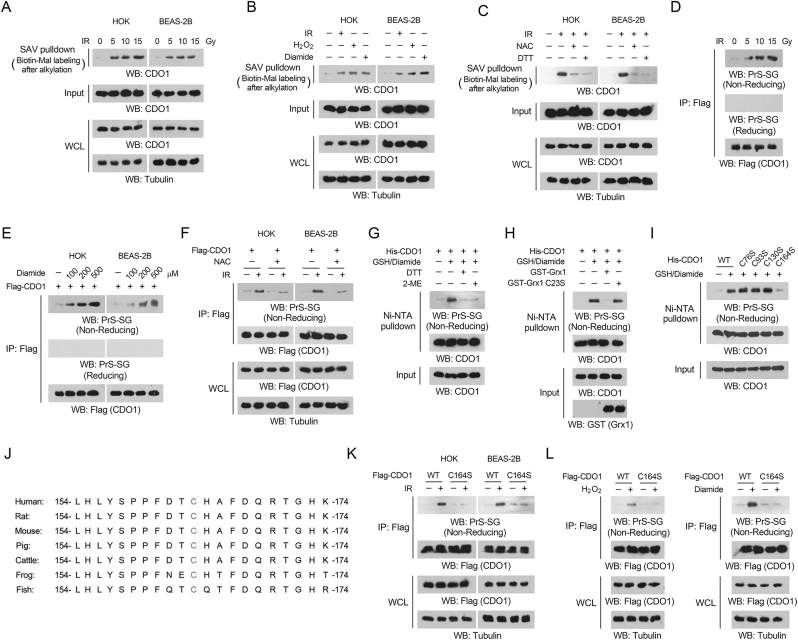
Ionizing radiation-induced oxidative stress promotes CDO1 glutathionylation at C164 (A-I, K, L) Immunoblotting analyses were performed utilizing the specified antibodies. If not specified, the immunoblotting analyses were conducted under reducing conditions. (A) HOK and BEAS-2B cells were subjected to radiation treatment at specified doses. 1 h after irradiation, cells were harvested and lysed, and the protein samples were treated with 100 mM maleimide, and precipitation was achieved by the addition of five volumes of acetone that had been pre-equilibrated at −20 °C, followed by air-drying. The resulting pellet was then resuspended in a solution containing 10 mM DTT and 0.1 mM biotin-maleimide to reduce any remaining oxidized cysteines, facilitating their reaction with biotin-maleimide. A streptavidin pulldown assay was subsequently conducted. WCL, whole cell lysate; SAV, streptavidin. (B) HOK and BEAS-2B cells were treated with 10 Gy of radiation (cells harvested 1 h after irradiation), or incubated with 500 μM H_2_O_2_ or 250 μM diamide for 30 min, and then the redox status of CDO1 was assessed. (C) HOK and BEAS-2B cells were pretreated with or without 10 mM NAC for 2 h prior to 10 Gy of radiation, or since 30 min post-irradiation the cells were treated with 2 mM DTT for another 30 min. 1 h after irradiation, the redox status of CDO1 was evaluated. (D) HOK cells expressing Flag-CDO1 were exposed to radiation at specified doses. 1 h after irradiation, Immunoprecipitation was then performed using the anti-Flag M2 antibody. (E) HOK and BEAS-2B cells expressing Flag-CDO1 were treated with diamide at indicated concentrations for 30 min. Immunoprecipitation was then performed using the anti-Flag M2 antibody. (F) HOK and BEAS-2B cells expressing Flag-CDO1 were pretreated with 10 mM NAC before 10 Gy radiation. 1 h after irradiation, immunoprecipitation was performed using the anti-Flag M2 antibody. (G) Purified His-CDO1 protein was incubated with 1 mM GSH and 0.5 mM diamide for 30 min. A Ni-NTA pulldown was performed, and the resulting precipitates were subsequently incubated with either 2 mM DTT or 2-ME. (H) Purified His-CDO1 protein was incubated with 1 mM GSH and 0.5 mM diamide for 30 min. A Ni-NTA pulldown was performed, and precipitates were incubated with either purified WT or GST-Grx1 C23S mutant protein for deglutathionylation. (I) Purified WT His-CDO1 protein or indicated mutant His-CDO1 protein was incubated with 1 mM GSH and 0.5 mM diamide for 30 min. A Ni-NTA pulldown was performed. (J) The adjacent sequences surrounding CDO1 C164 were aligned for the designated species, with C164 highlighted in red. (K) HOK and BEAS-2B cells expressing either WT Flag-CDO1 or Flag-CDO1 C164S were treated with 10 Gy of radiation, and 1 h after irradiation, immunoprecipitation was performed using the anti-Flag M2 antibody. (L) HOK cells expressing either WT Flag-CDO1 or Flag-CDO1 C164S were treated with 500 μM H_2_O_2_ (left panel) or 250 μM diamide (right panel) for 30 min, with immunoprecipitation conducted using the anti-Flag M2 antibody.

To determine the modification site, substitution of each cysteine residue with serine demonstrated that only mutation of C164 largely inhibited ionizing radiation-induced disulfide bond formation in CDO1 protein in both HOK and BEAS-2B cells (Supplementary Fig. S2E). Consistently, C164S, but not other mutation, markedly abolished diamide-mediated glutathionylation on purified CDO1 protein (Fig. 2I). This was in line with previous structural biology study showing undefined strong density against the thiol group of C164, hinting that this site might be susceptible to covalent redox modification [54]. Further, alignment analyses indicated that the sequences surrounding CDO1 C164 are highly conserved throughout evolution (Fig. 2J). Consistent with the aforementioned findings from *in vitro* assays, the glutathionylation of CDO1 induced by radiation, H_2_O_2_, or diamide in HOK or BEAS-2B cells was significantly diminished by the C164S mutation (Fig. 2K-L). These findings imply that CDO1 undergoes glutathionylation at the C164 site in response to oxidative stress induced by ionizing radiation.

### 3.3 C164 glutathionylation inhibits CDO1 by impairing CDO1 binding with substrate cysteine

To investigated the impact of C164 glutathionylation on the enzymatic activity of CDO1, WT His-CDO1 and His-CDO1 C164S proteins were incubated with GSH and diamide, and we found that CDO1 C164 glutathionylation led to a 60 % reduction in the activity of WT CDO1 protein (Fig. 3A). Although the C164S mutation itself could lead to a 20 % decrease in CDO1 activity, which aligns with previous reports [54], incubation with GSH and diamide did not cause further changes (Fig. 3A). Notably, the C164S mutation prevented the reduction in CDO1 activity in both irradiated HOK and BEAS-2B cells (Fig. 3B), indicating that C164 glutathionylation inhibits CDO1 activity.

**Fig. 3:**
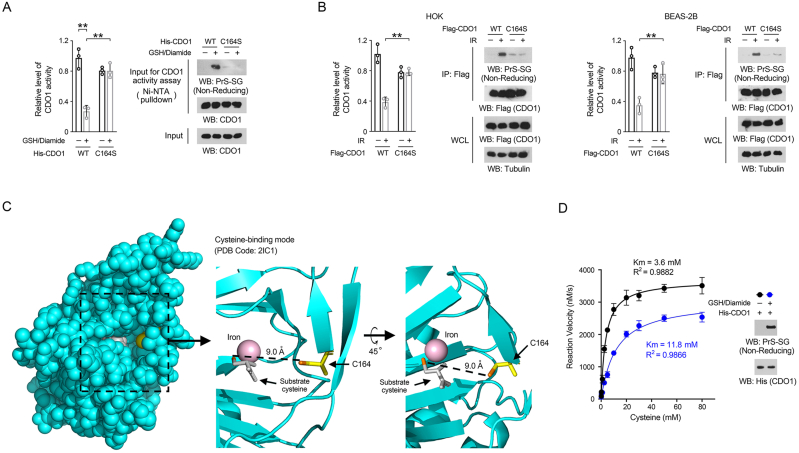
C164 glutathionylation inhibits CDO1 by impeding CDO1 binding with substrate cysteine (A, B, D) Immunoblotting analyses were performed utilizing the designated antibodies. (A) Purified WT His-CDO1 or His-CDO1 C164S proteins were incubated with 1 mM GSH and 0.5 mM diamide for 30 min. Ni-NTA pulldown assays were conducted, and the CDO1 activity within the precipitates was assessed. ∗∗*P* < 0.01. (B) HOK and BEAS-2B cells expressing either WT Flag-CDO1 or Flag-CDO1 C164S were subjected to 10 Gy radiation. 1 h after irradiation, immunoprecipitation was performed using anti-Flag M2 antibodies, and the CDO1 activity in the resulting precipitates was evaluated. Results are presented as mean ± standard deviation from three replicates. ∗∗*P* < 0.01. (C) The catalytic domain of human CDO1 protein (PDB code: 2IC1) is boxed (left panel), while the spatial arrangement of substrate cysteine (grey with its thiol group in orange), iron (purple) and C164 (side chain shown in yellow with its thiol group in orange) is shown in two different angles (right panel). The protein structural data were analyzed using PyMOL software. (D) Purified WT His-CDO1 protein was immobilized on beads, and was incubated with or without 1 mM GSH and 0.5 mM diamide for 30 min to introduce C164 glutathionylation. The CDO1 protein was precipitated, and the Km value for cysteine was measured.

According to the reported protein structure of human CDO1 (PDB code: 2IC1), one molecule of substrate cysteine and an iron molecule are visible within the catalytic domain. C164 and the substrate cysteine showed no obvious contact with each other, and the thiol group of the substrate cysteine is 9.0 Å away from the thiol group of the C164 (Fig. 3C) [20]. Strikingly, C164 is located at the entrance to the cysteine-binding site (Fig. 3C). Molecular dynamics (MD) simulation revealed that C164 glutathionylation may interfere the access of substrate cysteine to its binding site (Supplementary Fig. S3A–S3C). To test the impact of C164 glutathionylation on the cysteine-binding affinity of CDO1, we determined the Km value of CDO1 by measuring the CDO1 catalytic activity at various cysteine concentrations. We found that the Km value for CDO1 without glutathionylation was 3.6 mM, which was consistent with previous reports [19,54]. In contrast, the Km value for glutathionylated CDO1 was 11.8 mM (Fig. 3D). These results suggest that C164 glutathionylation diminishes CDO1 activity by impairing its binding to the substrate cysteine.

### 3.4 CDO1 C164 glutathionylation sustains cellular redox homeostasis and supports cell viability under ionizing radiation

To investigate the manipulation of CDO1 C164 glutathionylation in our cellular models, we knocked down endogenous CDO1 using specific short hairpin RNA (shRNA), followed by the expression of exogenous WT Flag-CDO1 or Flag-CDO1 C164S at comparable levels (Fig. 4A). The shRNA utilized targets a non-coding region of the CDO1 mRNA, ensuring that the sequence of interest is absent in the mRNA corresponding to the exogenous CDO1, thereby preventing any interference with its expression. We observed a significant induction of CDO1 glutathionylation in HOK and BEAS-2B cells expressing WT Flag-CDO1, as opposed to those expressing Flag-CDO1 C164S, following radiation exposure (Fig. 4B). Utilizing ^14^C-cystine as a tracer, we found that the production of ^14^C-pyruvate was diminished under radiation in cells expressing WT CDO1, which was largely prevented in cells expressing the CDO1 C164S mutant (Fig. 4C). This finding indicates that CDO1 C164 glutathionylation is essential for the radiation-induced repression of cysteine oxidation, though C164S mutation itself exhibited some opposite effects in non-irradiated conditions. Accordingly, the CDO1 C164S mutation significantly mitigated the ionizing radiation-induced synthesis of total GSH (Fig. 4D) and further lowered the GSH/GSSG ratios in HOK and BEAS-2B cells (Fig. 4E). Additionally, cells expressing CDO1 C164S exhibited heightened signals for 2,7-Dichlorofluorescein Diacetate (DCFDA) and 8-hydroxydeoxyguanosine (8-OHdG) under ionizing radiation, indicating increased levels of ROS and guanosine oxidation-related DNA damage, respectively (Fig. 4F-G) [55,56]. Ionizing radiation-induced oxidative stress may persist for longer than 24 h [57]. Accordingly, increased COD1 glutathionylation could be observed until at least 48 h after ionizing radiation, companied with increased expression level of glutamate cysteine ligase (GCL) (Supplementary Fig. S4A). Similar with that at 4 h post-irradiation, CDO1 C164S mutation also resulted in a declined GSH/GSSG ratio and an enhanced level of 8-OHdG 24 h after ionizing radiation (Supplementary Fig. S4B–S4C). Further, bromodeoxyuridine (BrdU) incorporation assay (Fig. 4H) and colony formation assay (Fig. 4I) revealed that, while the proliferative capacity was suppressed in both WT and mutant cells following ionizing radiation, it declined to a significantly lower level in cells expressing CDO1 C164S (Fig. 4H-I). These findings suggest that oxidative stress-induced CDO1 C164 glutathionylation is crucial for sustaining redox homeostasis and supporting cellular proliferation in the context of ionizing radiation.

**Fig. 4:**
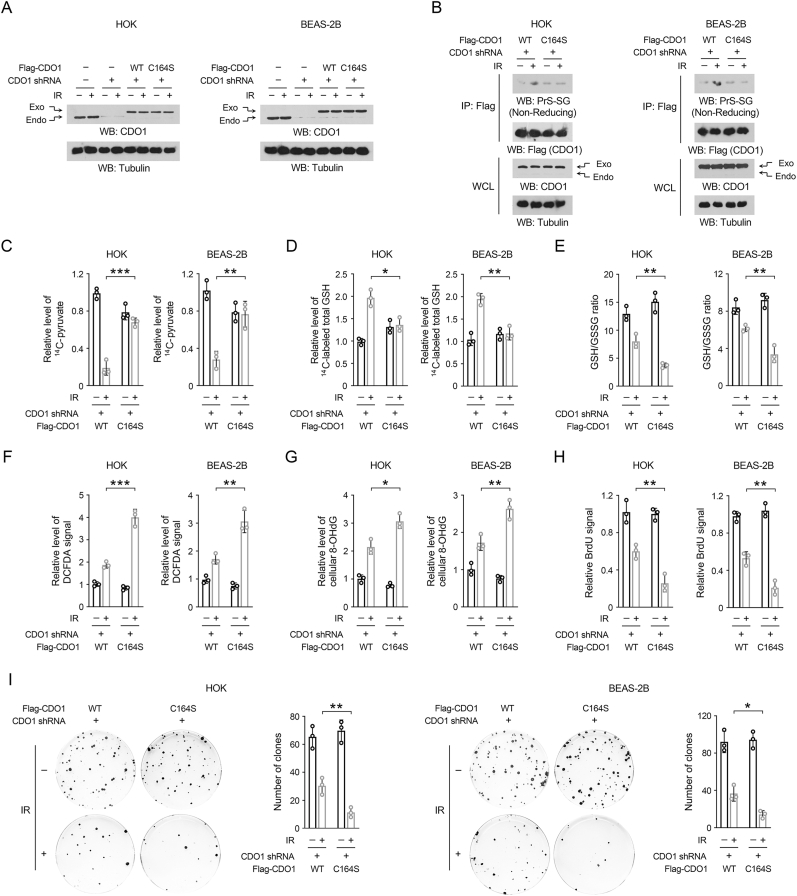
CDO1 C164 glutathionylation sustains cellular redox homeostasis and supports cell viability under ionizing radiation (A–H) The stable expression of CDO1 shRNA, WT Flag-CDO1, or Flag-CDO1 C164S was achieved in HOK or BEAS-2B cell lines. The shRNA was designed to specifically target the non-coding region of CDO1 mRNA. Cells were treated with 10 Gy radiation followed by specified measurements. (A) 1 h after irradiation, immunoblot analyses were performed utilizing the specified antibodies. (B) 1 h after irradiation, immunoprecipitation with the anti-Flag M2 antibody was performed. (C–D) 1 h after irradiation, cells were incubated with [1, 2, 1′, 2′-^14^C]-cystine (0.1 μCi/ml) for 30 min. The level of ^14^C-pyruvate (C) and total ^14^C-GSH (D) were subsequently quantified. Results are presented as mean ± standard deviation from three replicates. ∗∗*P* < 0.01, ∗∗∗*P* < 0.001. (E–G) 4 h after irradiation, the levels of the GSH/GSSG ratio (E), DCFDA (F) and 8-OHdG (G) were measured. Results are presented as mean ± standard deviation from three replicates. ∗*P* < 0.05, ∗∗*P* < 0.01, ∗∗∗*P* < 0.001. (H) 12 h after irradiation, the levels of BrdU signal were measured. Results are presented as mean ± standard deviation from three replicates. ∗∗*P* < 0.01. (I) After irradiation, cells were continuously cultured for 14 days for colony formation assay. Results are presented as mean ± standard deviation from three replicates. ∗*P* < 0.05, ∗∗*P* < 0.01.

## 4 Discussion

Amino acids are essential components of mammalian cells, functioning as substrates for protein synthesis and participating in a variety of biological processes [[58], [59], [60], [61]]. Among these amino acids, cysteine, which contains a unique thiol group, is particularly important due to its role as the primary precursor for the GSH. In the current study, we demonstrate that the enzymatic activity of CDO1 was impaired after exposure to ionizing radiation. Accordingly, by using ^14^C-cystine as a tracer, radiation treatment led to a significant reduction of ^14^C-pyruvate, an increase in cellular cysteine levels and an accumulated ^14^C-labeled total GSH, without an obvious change for cystine uptake, suggesting a repression of cysteine consumption through oxidation and a concomitant enhancement of GSH synthesis. Therefore, our findings indicate that CDO1 may represent the metabolic switch to fine-tune the two cysteine-utilizing routes under stressful conditions.

Glutathionylation represents a post-translational modification characterized by the formation of mixed disulfides between GSH and specific cysteine thiols within proteins, particularly under conditions of oxidative stress [62,63]. In the current study, we identify CDO1 as a novel protein substrate for glutathionylation. We found that CDO1 with oxidized cysteine was accumulated under radiation-induced oxidative stress. This modification was reversible, as it was abolished in the presence of the reducing agent NAC or DTT, and could be also induced by the treatment with either peroxide H_2_O_2_ or thiol oxidant diamide even in the non-irradiated cells. By using DYn-2, we also detected an increased level of sulfenylation for CDO1 protein, which is in line with previous report showing that sulfenylation is intrinsically unstable and functions as an intermediate prior to forming glutathionylation [64].

By mutation of each cysteine residue, we revealed that C164 was the major site undergoing glutathionylation. A previous study solved the crystal structure of rat CDO1 protein and found an unknown density against the thiol group of Cys164, suggesting that Cys164 was likely covalently linked with a potential ligand through a disulfide bond [65]. Consistent with this report, the present data demonstrates that CDO1 was glutathionylated under ionizing radiation, by which Cys164 was linked with a GSH molecule. Our data is also in line with bioinformatic analyses results from ReDisulphID website (https://redisulphid.net/Q16878.html), which indicated that disulfide bond formation on other site of CDO1, including C93 and C130, are unlikely events. Though CDO1 binds to a substrate cysteine, the thiol group of the bound substrate cysteine was pointing towards the opposite direction from the C164, and the distance between the thiol group of C164 and the thiol group of the bound substrate cysteine is as far away as 9.0 Å, hinting that it was not likely to form a disulfide bond between the bound cysteine and C164.

Glutathionylation introduces a covalently linked GSH molecule, which frequently results in protein conformational change, either locally or globally, thereby influencing the activity, stability, or subcellular localization of the modified proteins [16]. In the present data, modeling analyses suggest that the covalently linked glutathionyl group likely masks the entrance, thereby probably blocking the access of substrate cysteine to the catalytic site of CDO1. Biochemical analyses further showed that C164 glutathionylation reduced the binding affinity between CDO1 and substrate cysteine, revealed by the apparently increased Km value, and declined CDO1 enzymatic activity. In contrast, the CDO1 C164S mutation, which is resistant to glutathionylation and largely retains CDO1 enzymatic activity under ionizing radiation, significantly impairs radiation-induced GSH synthesis, coinciding with an apparent increase in cellular ROS levels. Expression of CDO1 C164S in both oral and lung epithelial cells further diminished cell viability after exposure to radiation. Interestingly, our data shows that CDO1 C164S mutation resulted in a further declined GSH/GSSG ratio and a further enhanced level of 8-OHdG at both 4 h and 24 h after ionizing radiation, companied with basal or enhanced GCL expression level, respectively. This data further supports that COD1 C164 glutathionylation has an important impact on cellular redox homeostasis and cell viability under either short- or long-term oxidative stress induced by ionizing radiation.

**Fig. 5:**
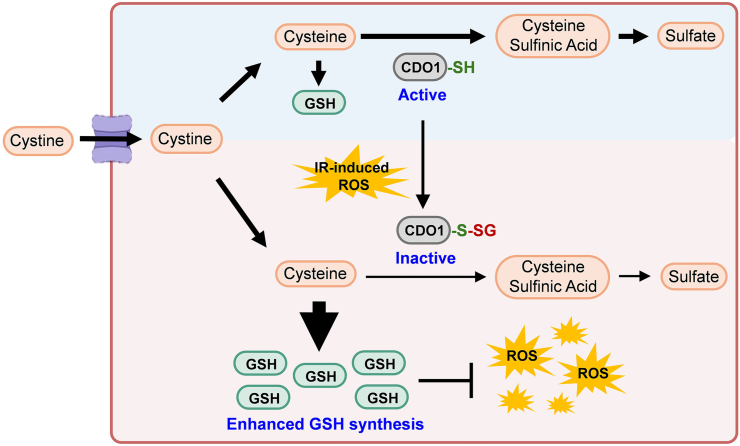
A schematic of redox regulation of CDO1 under ionizing radiation-induced oxidative stress Ionizing radiation promotes the glutathionylation of CDO1, leading a reduced enzymatic activity of CDO1, which results in the suppression of the cysteine oxidation pathway and an increase in the synthesis of GSH. This molecular event supports cell viability by preserving redox homeostasis under ionizing radiation.

In summary, the present data reports a novel protein redox modification-based mechanism under ionizing radiation (Fig. 5). Oxidative stress-induced CDO1 C164 glutathionylation reduces the substrate-binding affinity and enzymatic activity of CDO1, leading to reduced cysteine oxidation with enhanced GSH synthesis. This molecular mechanism sustains cellular redox homeostasis, which benefits cell viability upon ionizing radiation-mediated damages.

## CRediT authorship contribution statement

**Yumin He:** Investigation, Formal analysis, Data curation. **Dan Li:** Methodology, Investigation, Data curation. **Hongping Ye:** Methodology, Investigation, Data curation. **Jiang Zhu:** Conceptualization, Methodology, Writing – review & editing. **Qianming Chen:** Conceptualization, Funding acquisition, Writing – review & editing. **Rui Liu:** Writing – original draft, Conceptualization, Funding acquisition, Writing – review & editing.

## Funding

This work was supported by 10.13039/501100001809National Natural Science Foundation of China program 82330029 (Q.C.), Sichuan Science and Technology Program 2023NSFSC1924 (R.L.), and Research Funding from 10.13039/100020756West China School/Hospital of Stomatology Sichuan University RD-03-202404 (R.L.) and RCDWJS2023-2 (R.L.).

## Declaration of competing interest

The authors declare that they have no known competing financial interests or personal relationships that could have appeared to influence the work reported in this paper.

## Figures and Tables

**figs1:**
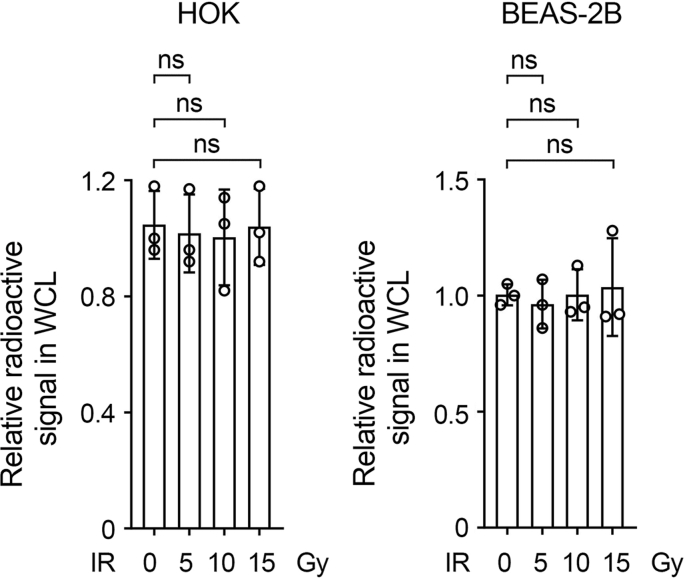


**figs2:**
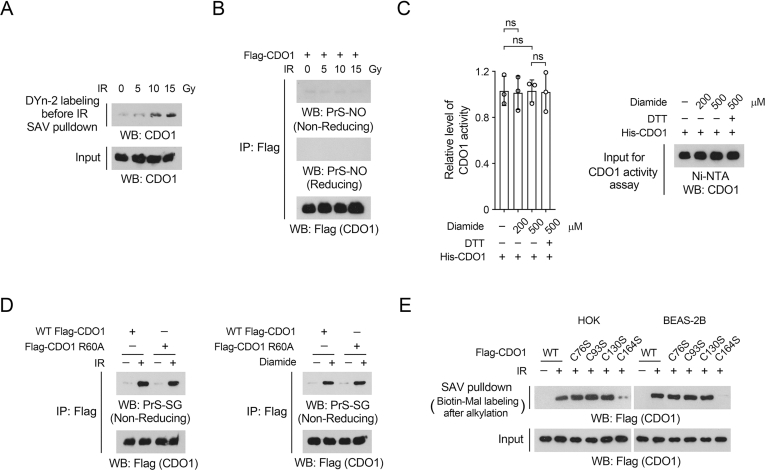


**figs3:**
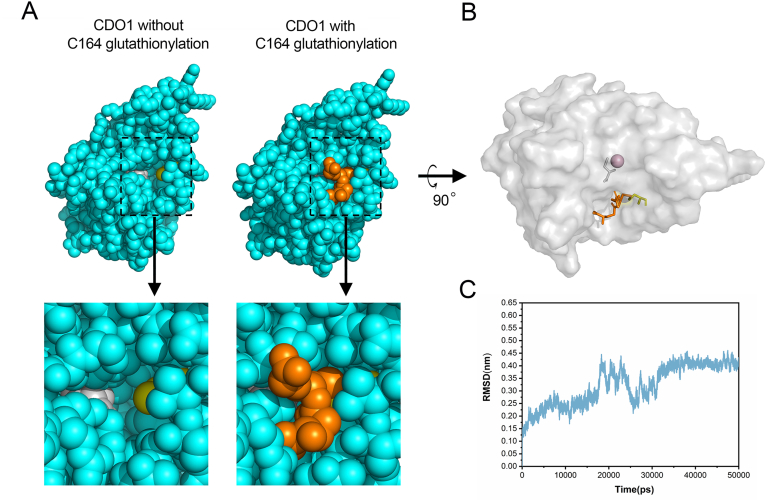


**figs4:**
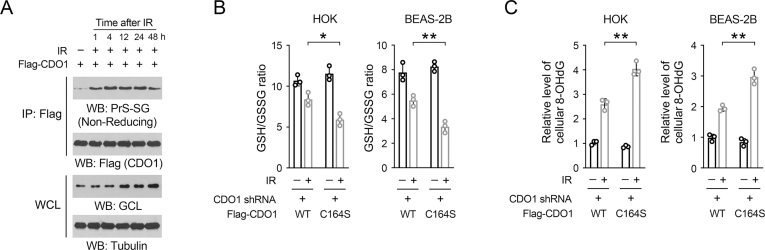


## Data Availability

Data will be made available on request.
