# Supplementary material for: Oxidative stress-induced CDO1 glutathionylation regulates cysteine metabolism and sustains redox homeostasis under ionizing radiation
Source: Redox Biol. 2025 Apr 30;83:103656. doi: 10.1016/j.redox.2025.103656 (PMC12146662; doi:10.1016/j.redox.2025.103656)
Supplement: Multimedia component 1 [file mmc1.docx]

**Supplementary figure legends**

**Figure S1. Radiation-induced oxidative stress inhibits CDO1 activity**

HOK and BEAS-2B cells were treated with radiation at indicated doses. 1 h after irradiation, cells were incubated with [1, 2, 1’, 2’-^14^C]-cystine (0.1 μCi/ml) for 10 min and washed. The radioactive signal level was evaluated in the WCL from cells. The data was shown as mean ± SD from three replicates. ns, not significant. WCL, whole cell lysate.

**Figure S2.** **CDO1 was glutathionylated at C164 under radiation-induced oxidative stress**

(A-E) Immunoblotting analyses were performed utilizing the designated antibodies.

(A) The cells were incubated with 5 mM DYn-2 probe for 30 min, and treated with radiation at indicated doses. The cell lysates were subjected to streptavidin pulldown, and the precipitates were analyzed by immunoblot with anti-CDO1 antibody.

(B) HOK cells expressing Flag-CDO1 were exposed to radiation at specified doses, and 1h after irradiation, immunoprecipitation was performed using the anti-Flag M2 antibody.

(C) Purified CDO1 protein was incubated with 200 or 500 mM diamide for 30 min, and Ni-NTA pulldown was performed. The precipitates were treated with or without 5 mM DTT for 1 h. The CDO1 activity in the precipitates were examined. Results are presented as mean ± standard deviation from three replicates. ns, not significant.

(D) HOK cells with expression of WT Flag-CDO1 or Flag-CDO1 R60A were exposed to 10 Gy radiation (left panel, cells harvested 1 h after irradiation) or incubated with 250 µM diamide for 30 min (right panel). Immunoprecipitation was performed using the anti-Flag M2 antibody.

(E) HOK and BEAS-2B cells with expression of WT Flag-CDO1 or mutant Flag-CDO1 were exposed to 10 Gy radiation. 1h after irradiation, the redox status of CDO1 was assessed.

**Figure S3. Molecular dynamics simulation of CDO1 with C164 glutathionylation**

(A) Molecular dynamics simulation of CDO1 with C164 glutathionylation was performed based on the reported CDO1 protein structure (PDB code: 2IC1). The catalytic domain of CDO1 with (right panel) or without (left panel) C164 glutathionylation were boxed, and enlarged. The substrate cysteine (shown in grey), C164 (shown in yellow), and the glutathionyl group (shown in orange) were annotated with different colors.

(B) CDO1 with C164 glutathionylation is shown with transparent protein surface. The substrate cysteine (shown in grey), C164 (shown in yellow), iron (shown in purple) and the glutathionyl group (shown in orange) were annotated with different colors.

(C) The RMSD for CDO1 with C164 glutathionylation converged after 35 ns. CDO1 protein structure analyses were performed based on equilibrated trajectory ensemble (35-50 ns).

**Figure S4. CDO1 C164 glutathionylation sustains cellular redox homeostasis**

(A) HOK cells with expression of Flag-CDO1 were treated with 10 Gy ionizing radiation. After indicated time, immunoprecipitation was performed using anti-Flag M2 antibodies,

(B-C) The stable expression of CDO1 shRNA, WT Flag-CDO1, or Flag-CDO1 C164S was achieved in HOK or BEAS-2B cell lines. Cells were treated with 10 Gy radiation. 24 h after irradiation, the levels of the GSH/GSSG ratio (B), and 8-OHdG (C) were measured. Results are presented as mean ± standard deviation from three replicates. **P* < 0.05, ***P* < 0.01.
