# Supplementary material for: Oxidative stress-induced CDO1 glutathionylation regulates cysteine metabolism and sustains redox homeostasis under ionizing radiation
Source: Redox Biol. 2025 Apr 30;83:103656. doi: 10.1016/j.redox.2025.103656 (PMC12146662; doi:10.1016/j.redox.2025.103656)

Supplementary Figure: uncropped WB images

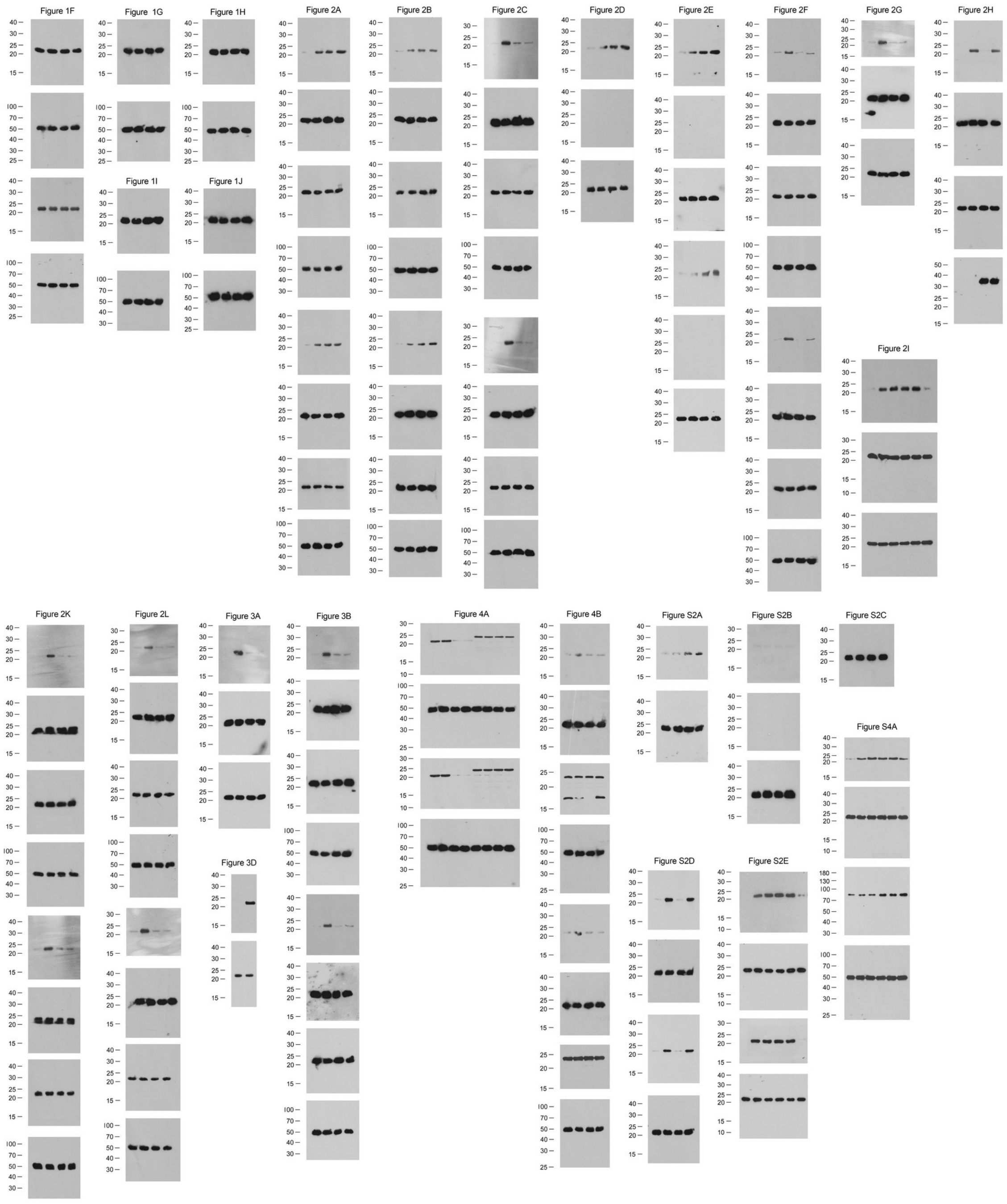

Supplement: Supplementary file 2 [file mmc2.docx]
